# Validation of Architecture Effectiveness for the Continuous Monitoring of File Integrity Stored in the Cloud Using Blockchain and Smart Contracts

**DOI:** 10.3390/s21134440

**Published:** 2021-06-29

**Authors:** Alexandre Pinheiro, Edna Dias Canedo, Robson de Oliveira Albuquerque, Rafael Timóteo de Sousa Júnior

**Affiliations:** 1 Electrical Engineering Department, National Science and Technology Institute on Cybersecurity, University of Brasília (UnB), P.O. Box 4466, Brasília 70910-900, Brazil; robson@redes.unb.br (R.d.O.A.); desousa@unb.br (R.T.d.S.J.); 2 Department of Computer Science, University of Brasília (UnB), P.O. Box 4466, Brasília 70910-900, Brazil; ednacanedo@unb.br

**Keywords:** blockchain, cloud computing, data security, smart contracts, trust

## Abstract

The management practicality and economy offered by the various technological solutions based on cloud computing have attracted many organizations, which have chosen to migrate services to the cloud, despite the numerous challenges arising from this migration. Cloud storage services are emerging as a relevant solution to meet the legal requirements of maintaining custody of electronic documents for long periods. However, the possibility of losses and the consequent financial damage require the permanent monitoring of this information. In a previous work named “Monitoring File Integrity Using Blockchain and Smart Contracts”, the authors proposed an architecture based on blockchain, smart contract, and computational trust technologies that allows the periodic monitoring of the integrity of files stored in the cloud. However, the experiments carried out in the initial studies that validated the architecture included only small- and medium-sized files. As such, this paper presents a validation of the architecture to determine its effectiveness and efficiency when storing large files for long periods. The article provides an improved and detailed description of the proposed processes, followed by a security analysis of the architecture. The results of both the validation experiments and the implemented defense mechanism analysis confirm the security and the efficiency of the architecture in identifying corrupted files, regardless of file size and storage time.

## 1. Introduction

The large-scale adoption of electronic document management systems (EDMSs) in public administration agencies and private companies has replaced the elaboration and processing of documents in a physical medium (paper) with exclusively electronic documents. Currently, most countries have enacted laws that support the use of electronic documents, such as [1] in Brazil and [2] in the United States of America, making them legally equivalent to manually signed documents.

As a result, legislation such as [3] in Brazil and [4] in the United Kingdom, whose function is to delimit the validity, temporality, and confidentiality classification, also applies to electronic documents. Temporality, a characteristic that determines the minimum document storage and preservation time, may range from 1 to more than 30 years, depending on the country and the nature and content of the document.

Once a document is processed, the need to access it is rare, especially more than a year after it is filed. Depending on the flow of documents processed in an organization, the size of the backup copies of documents for a period of one year can easily exceed ten gigabytes.

Cloud computing, with its emphasis on storage services, presents as an economical and easy-to-manage solution for storing data for long periods as required by legislation regarding electronic documents. However, the outsourcing of storage services faces the risk of the stored data being leaked, corrupted, or simply deleted by the service provider.

The monitoring of the integrity of files stored in the cloud has been the subject of several studies [5,6,7,8,9], which adopted different mechanisms and technologies. Pinheiro et al. [10] presented a protocol designed for the periodic monitoring of the integrity of large stored files based on computational trust and symmetric cryptography. Aiming to enhance the referred protocol, improve its efficiency, and increase its security mechanisms, Pinheiro et al. [11] proposed a new architecture that upgraded the protocol proposed in the previous research integrating it with a solution based on blockchain and smart contracts technologies. Notably, this architecture was designed to allow its integration with proprietary solutions adopted by the main cloud service providers (CSPs), such as Google Cloud [12], AWS [13], and Azure [14]. As can be seen in the CSP application proposal presented in [11], among the characteristics that facilitate its integration, we highlight that the architecture requires only well-known software components and privileges processes whose communication between participants is asynchronous.

Based on the above, this paper presents a detailed step-by-step description of the process that composes the architecture proposed in [11]; a security analysis; and a validation of the applicability, effectiveness, and efficiency of this architecture when applied to the monitoring of the integrity of large files stored in the cloud for long periods.

## 2. Background

This section presents a brief description of important concepts and technologies adopted in this research study, such as distributed ledger technology, blockchain, smart contracts, and the solidity programming language.

### 2.1. Distributed Ledger Technology

Ledgers have always been the core of commerce, being used to record many things, mainly assets such as money and properties. Information was initially recorded on clay tablets, evolving to papyrus and parchment, then to paper, from where it moved to the computing environment in the form of bytes [15]. According to Hill et al. [16], a ledger is a record of economic transactions that includes, amongst other things, money, accounts receivable, inventories, permanent assets, accounts payable, accumulated expenses, debts, capital holdings, equity, revenue, costs, wages, and depreciation; in other words, it is the book in which all accounts are recorded and maintained.

A distributed ledger is essentially an asset database that can be shared through a composite network of multiple sites, countries, or institutions, in which an identical copy of a ledger is maintained, and any change is reflected in all copies in a few minutes, or even seconds [15]. Moreover, by assuming the existence of malicious nodes, distributed ledgers require the application of a consensus mechanism to withstand Byzantine failures such as incorrect data storage, whose distributed execution across nodes provides multiple points of authenticity for each transaction that has occurred. The consensus mechanisms are designed to negotiate and reach an agreement about the state of the replicated data stored in each node [17].

According to Lange et al. [18], distributed ledger technology (DLT) is the designation for a database type that is shared through a point-to-point network; whose transaction data are synchronized between network nodes, stored immutably, and protected using cryptographic techniques; and where decisions in the network are managed by consensus algorithms. In Fernando and Ranasinghe [19], a DLT network was described as a collection of interconnected nodes, where each node maintains a copy of the database, called a ledger.

### 2.2. Blockchain

According to Maull et al. [20], blockchain technology is a particular type of DLT. In Lange et al. [18], blockchain was described as a distributed, chronological, immutable, and synchronized ledger shared over a point-to-point network; based on transactions that are individually executed and recorded using consensus algorithms; and finally grouped and stored in sets of transactions called blocks, interconnected with each other through cryptographic techniques. In another definition, Walport [15] described blockchain as a type of database in which a set of records are inserted into a block that is chained with the next block using a cryptographic signature, which can be shared and corroborated by anyone who has the appropriate permission.

According to Ølnes et al. [21], blockchain technology has the following characteristics: the same information is stored in different network nodes forming a distributed ledger; new data are included only when the nodes reach a consensus, preventing any removal after inserted, allowing each node to track its history. These characteristics reduce the dependence on a central actor, the chance of manipulation, and the risk of system failures since all nodes have a complete set of information. According to Alharby and van Moorsel [22], blockchain technology allows transactions to be completed directly between the parties, without the need for intervention by a trusted third party, and once a new block is inserted into the blockchain, transactions registered in this block cannot be changed or reversed.

Blockchain technology allows two or more entities, whether they have a previous relationship of trust or not, to safely exchange values over the internet, without the need for the participation of a third party [23]. In practice, blockchain technology does not eliminate the need for trust but minimizes it and distributes it evenly over the network [16].

### 2.3. Smart Contracts

Smart contracts are digital contracts that are tamper-proof and generally self-enforce through automated execution [24]. A smart contract is a digital contract that imposes itself or becomes prohibitively expensive to break [25]. Each smart contract is a computer program that runs on blockchain in order to enforce the terms of a contract between untrusted parties [22].

The purpose of a smart contract is to facilitate the exchange of assets, such as goods and services of any kind, in an automated and conflict-free manner [24]. These assets are made available to all parties involved or only to certain parties whenever the previously defined rules are observed [22]. Since blockchain technology can be used as a distributed state machine without the need for a trusted third party, it provides a wholly suitable environment to support the execution of smart contracts [25].

A smart contract is developed as a script to be stored within the blockchain and has a unique access address, whose execution is activated whenever a transaction is assigned to the respective address. Its execution occurs automatically and independently in each of the network nodes using the data included in the transaction that triggered as parameters [26].

The behavior of smart contracts is predictable, and each contract operates as an autonomous actor within the blockchain. Both smart contract installation and execution are carried out as part of a blockchain transaction [22]. Among the characteristics of smart contracts, each contract has its own status, it can custody assets on the blockchain in the same way as users, it must describe all possible results, it must be deterministic (the same input always produces the same output), and its code can be inspected by any of the network participants [26].

### 2.4. Blockchain Platforms

Currently, there are several initiatives to implement blockchain platforms for diverse purposes, such as: Bitcoin [27,28], Ethereum [29,30], Namecoin [31,32], Multichain [33], Zcash [34], Monero [35,36], and the Hyperledger Fabric [37,38]. Among the reasons for these initiatives, we highlight two main obstacles to the adoption of blockchain platforms, namely high power consumption and the low transaction processing speed.

The efficiency of each platform (processing speed versus power consumption) is directly related to the adopted consensus mechanism. Platforms such as Bitcoin [27] and Ethereum [29] adopt the proof-of-work (PoW) consensus mechanism in their public networks, which is secure but inefficient [17]. Other platforms, such as Hyperledger Fabric [37], adopt more efficient but less secure consensus mechanisms. For this reason, these platforms require tighter control over the input and output of network participants, as well as over which of these participants will or will not participate in the consensus process.

After a preliminary analysis, we chose to detail the Ethereum and Hyperledger Fabric platforms. The popularity of these platforms [39] and the fact that several blockchain solution providers highlight them as the most used in the implementation of corporate solutions [40,41] contributed to this choice. Furthermore, the chosen platforms allowing the use of smart contracts to control assets with characteristics and operations different from those traditionally used in cryptocurrency management was key in this decision.

#### 2.4.1. Ethereum

According to Sajana et al. [42], Ethereum is an open platform that allows the construction and use of decentralized applications that adopt blockchain technology, designed to be flexible and adaptable, whose focus is the execution of smart contracts (programs) that move assets, which can each represent the ownership or possession of a good/asset. In Ethereum networks, miners work to earn cryptographic tokens, “Ether”, which are also used to pay for performed service fees/transactions.

The core of Ethereum is its virtual machine named Ethereum Virtual Machine (EVM), which runs on each node in the network and is extremely fault-tolerant. The EVM enables the decentralized execution of complex algorithms written in a friendly programming language and is responsible for maintaining consensus on the Ethereum network [42]. These algorithms are high-level abstractions that can be written in a programming language such as Solidity [43], and are called smart contracts. After the smart contract is compiled in the form of a bytecode specific to EVM, this bytecode is inserted into the blockchain network (BN) through a transaction [44].

In Ethereum, the order of transactions is critical to maintaining the consistency of the ledger, and all participants must reach a consensus on the order of all transactions that occurred on the network, regardless of whether or not they participated in a particular transaction. To protect the ledger against fraud attempts such as double-spending, Ethereum employs consensus mechanisms such as proof-of-work [45].

To avoid problems with abuses of the use of the computational resources of the network, all programmable calculations in Ethereum are subject to fees, the cost of which is variable and charged through a computational unit of work called “gas”. To calculate the fee in Ether (cryptocurrency), the gas system depends on three components: gas usage, gas price, and gas limit.

Gas usage is the amount of gas consumed in each transaction, which varies according to the complexity of the method executed in the contract used, the quantity and size of the input parameters, and the current status of the contract. The gas limit is the maximum amount of gas that a transaction can consume before being aborted, and the gas price is the amount in Ether that the transaction requester is willing to pay for each unit of gas consumed by the transaction, which may or may not arouse the interest of the nodes responsible for mining new blocks to insert the transaction in the ledger [46].

On the Ethereum public BN, the gas price fluctuates according to the value traded on the network at the instant each transaction is processed [47]. This feature makes it difficult to effectively estimate the cost of transactions over time. For this reason, the use of a private BN for cost optimization purposes should be considered.

#### 2.4.2. Hyperledger Fabric

Hyperledger is a Linux Foundation project that supports collaborative and open-source distributed ledger solutions based on blockchain. The project’s main objectives are the improvement in the performance and reliability of these solutions, and the advancement of collaboration between industries. Among the frameworks hosted by the Hyperledger project are Fabric, Burrow, Iroha, Sawtooth, and Indy [42].

Androulaki et al. [38] defines Hyperledger as an open-source, modular, and extensible system for the implementation and operation of licensed blockchain networks. It is an extensible system that enables distributed applications to run and supports modular consensus protocols, which can be tailored to particular trust models and use cases. The system securely records your transaction history through a ledger replicated between the participating nodes, which accepts only inclusions and has no embedded cryptocurrency.

According to Sajana et al. [42], Hyperledger Fabric is a distributed ledger platform for the execution of programs called chaincode (smart contracts), with a modular architecture that delivers a high degree of resilience, confidentiality, and flexibility. A Hyperledger Fabric network is a licensed network where transactions are private, confidential, and auditable, and whose participants must be previously registered.

The Hyperledger Fabric introduces a new blockchain architecture to provide resilience, flexibility, scalability, and confidentiality. Its architecture allows the execution of distributed applications written in general-purpose programming languages without the systemic dependence of a native cryptocurrency. In addition, the Fabric architecture separates the transaction flow into three stages, whose executions can be performed by different entities: transaction execution and verification of its correctness, ordering of transactions using a consensus algorithm, and validation of transactions from application-specific trust assumptions [38].

Obtaining consensus in Hyperledger Fabric is a process that encompasses the transaction flow as a whole, which starts when the transaction is proposed to the network and completes only when inserted in the ledger. Nodes have different functions and tasks in the consensus process according to the role they play. These roles are divided into clients, peers, and orderers. The clients create and invoke the transactions. The peers keep the ledger and receive messages ordered from the orderers to register new transactions. The orderers provide the communication channel between the clients and the peers over which they distribute the messages containing the transactions [45].

### 2.5. Solidity

Solidity is a high-level, object-oriented programming language designed to implement smart contracts for the Ethereum platform [43]. Smart contracts developed in Solidity are compiled and transformed into bytecodes for exclusive execution in Ethereum virtual machines. The Solidity language provides the tools for smart contracts to exchange messages with each other, receive and transfer virtual currency (Ether), and to define the fee (amount of gas) that a contract is willing to pay for processing transactions requested to other smart contracts [48].

Another resource provided by the Solidity language is the use of events that can be arbitrarily generated by smart contracts, with each event containing a name, the address of the source contract, and any number of parameters [49]. The events are recorded in data structures called transaction logs, which are incorporated into the blockchain. These logs cannot be accessed by smart contracts, and must be monitored by applications through the Ethereum client [43].

## 3. Related Works

This section presents recent research related to the monitoring of the integrity of files stored in the cloud, and the use of solutions based on blockchain and smart contracts.

### 3.1. Monitoring the Integrity of Files Stored in the Cloud

In Amaral et al. [5], a new protocol is proposed called Hyper Scalability, Availability, Integrity Layer (Hy-SAIL), whose purpose is to guarantee the integrity and the ability to recover files stored in the cloud. Hy-SAIL achieves this purpose by applying a solution called Online Codes [50], which generates data blocks for error correction and distributes them among several storage providers.

These data blocks allow the recovery of corrupted parts of a file stored in a provider from the information stored in others. Hy-SAIL ensures file integrity through a periodic challenge–response-type checking mechanism based on the algebraic properties of finite fields (Galois field (GF)) and the generation of homomorphic message authentication codes.

In preparation for submitting the file content, Hy-SAIL divides it into *n* parts called message blocks from which the protocol generates the auxiliary blocks necessary for fail recovery. For challenge preparation, the protocol selects primary polynomials, and for each polynomial, it generates authentication codes for each message, which are stored by the file owner (client).

The file content is distributed randomly to storage providers in the form of fixed-size check blocks, containing the content of one or more message blocks (or auxiliaries), generated through the homomorphic XOR function, executed bit-by-bit on the content of each pair of blocks. To verify block integrity, the protocol sends challenges containing a polynomial and a block identifier. After receiving the challenge response, the protocol validates the referred response through the message authentication code stored by the client.

Zhao et al. [7] proposed a method for the public auditing of files stored in the cloud. The model adopts a skip list [51] based on classification, originally proposed by [52], as a data structure through which it supports dynamic operations such as inclusion, alteration, and exclusion of the stored data. The proposed method allows the owner (client) or a designated third party (auditor) to check the file integrity without compromising content privacy. This method also allows cloud storage services (CSSs) to prevent clients from dishonestly claiming loss of data because it implements a mechanism to issue receipts signed by both parties.

The method is characterized by storing only private data and verifying metadata on the client since the skip list is stored in the CSS. The protocol used by the model to verify file integrity divides the process into three movements called commitment, challenge, and response, executed respectively by the CSS, the auditor, and the CSS. The technique used is based on the compact recoverability tests proposed in [53] and its implementation is based on the homomorphic properties of the adopted algorithms. The method’s safety is directly related to the difficulty in solving the Diffie–Hellman computational problem [54] and in the discrete logarithm on bilinear groups.

Wang et al. [8] proposed a scheme for the dynamic verification of cloud-stored data ownership based on an identity called Identity-Based Non-Repudiable Dynamic Provable Data Possession (ID-NR-DPDP). This scheme uses a monotonic dynamic structure, i.e., ordered and liable, for comparison according to the moment of its generation called an index logic table (ILT), which allows the inclusion, alteration, and exclusion of blocks in previously stored files. ID-NR-DPDP defines the interaction among four entities: the file owner (client), the private key generator (PKG), the judge responsible for resolving possible differences, and the CSS, where both the PKG and the judge are considered trustworthy by all clients and the CSSs.

The PKG is responsible for generating the private keys for both the client and the CSS. The client uses their private key to sign the information generated by the client (block labels and data list) which, in turn, is sent by the client together with the file data for storage in the CSS. Meanwhile, the CSS uses its key to sign the receipt that confirms the file storage. In addition to the file data blocks (fractions), the labels calculated and signed by the client for each block, the data list containing a copy of the ILT, a name generated for the file, and a set of randomly chosen values used for calculations and the client signature are stored in the CSS.

In terms of security, ID-NR-DPDP implements the Diffie–Hellman [55] key exchange protocol to prevent tampering with the responses from the CSS and ILT to resist exclusion/insertion attacks, as named and demonstrated by the authors. The public key cryptography implemented in ID-NR-DPDP adopts the identity-based signature method proposed in [56], which allows authenticating the entities involved without the need to manage certificates or the use of public key infrastructure. In the proposed scheme, the client can perform data integrity verification or assign this responsibility to another entity named an auditor. The ID-NR-DPDP bases its security on the difficulty of solving the Diffie–Hellman [54] computational problem.

Jeong et al. [9] presented an effective data possession verification scheme based on the use of the Bloom Filter (BF) [57]. The developed schema imposes a consistency guarantee on large volumes of data generated by low computational devices (Internet of Things) and stored in cloud services. The proposed scheme divides the data to be cloud-stored into blocks, for which BFs are generated. These BFs are stored by the device that generated the data or by a trusted third party, which, in turn, through a challenge–response-type process, will be responsible for confirming the effective data possession by the storage service.

The BF is a data structure generated from the execution of hash algorithms on the content of the blocks applied to confirm whether the cloud-stored data content is compatible with the data that gave rise to the BF, with a determined error rate (false positives). The maximum acceptable error rate limit determines both the number of blocks and the BF size. According to the authors, the proposed solution is suitable for processing large amounts of data because it requires less processing time since it does not require any key generation mechanism, although false positives reduce the rate of failure identification. Furthermore, the proposed solution does not present significant differences concerning the rates obtained by other solutions based on homomorphic cryptography.

### 3.2. Blockchain and Smart Contracts

Zhang et al. [58] proposed a scheme for public verification of the integrity of cloud-stored files resistant to procrastinating auditors, without the use of certificates, named Certificateless Public Verification scheme against Procrastinating Auditors (CPVPA). The CPVPA uses a challenge–response model to verify integrity that adopts the aggregate signature technique based on the Diffie–Hellman [54] computational problem on certain elliptic and hyperelliptic curves proposed in [59].

The CPVPA provides the possibility of third parties (auditors) carrying out the integrity check. The resistance to procrastination, or the malicious performance of these auditors, is implemented using blockchain technology. For this, the auditors securely (unchangeably) record the information generated by each verification process execution in the blockchain for subsequent auditing by the file owner.

CPVPA’s cryptographic mechanism uses a third party as the authority responsible for generating partially private keys for users, a functionality that dispenses with the use of digital certificates based on public key infrastructure and, consequently, exempts CPVPA from the problems inherent in managing certificates such as revocation, storage, distribution, and verification. Since CPVPA was developed based on properties of the consensus’ algorithms based on PoW, as the maximum number of possible inconsistent blocks in the chain and the impossibility of predetermining the hash of a future block, the construction of CPVPA requires the adopted blockchain implementation to exclusively use PoW as its consensus mechanism.

Rahalkar and Gujar [60] proposed a model that integrates a decentralized file storage system for use in peer-to-peer (P2P) networks called InterPlanetary File System (IPFS) and blockchain technology to store and distribute data on the web, preserving data integrity and security in a reliable and fault-tolerant manner. The proposed model divides file content into parts and stores the parts on different nodes of the P2P network using IPFS, which, in turn, uses the Distributed Hash Table (DHT) protocol. To access individual file content, the model uses a name server called InterPlanetary Naming Server (IPNS), which maps the file name to the hash generated from its content. The model uses blockchain to store the metadata of each file, which contain its hash, which, in turn, is used by the model when the file is retrieved from the web to validate its integrity.

Meroni et al. [61] proposed carrying out continuous and autonomous monitoring of business processes involving non-automated activities (artifact-driven) performed in different parts, which adopts blockchain technology as a reliable and auditable means for exchanging data between business process participants. The authors investigated the effects generated by the choice between guaranteeing the storage of all data generated by monitoring and the minimization of these data for registration in the blockchain. Furthermore, they also evaluated the costs, in virtual currency, of the publication of these data on public blockchain networks. The proposal was validated on a hybrid platform using a prototype that uses smart contracts on the Ethereum Blockchain platform, integrated with the distributed file system, IPFS.

## 4. Architecture for Monitoring the Integrity of Files in the Cloud

**Figure 1:**
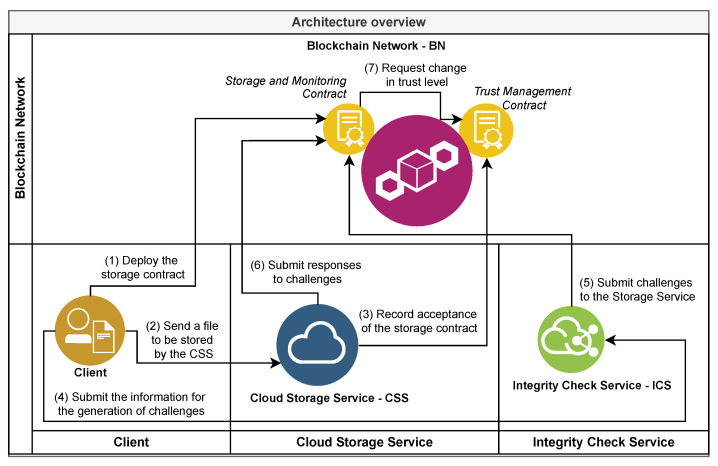
Architecture overview.

This architecture aims to provide a solution option for the secure and monitored storage of large files for long periods in unreliable cloud service providers (CSPs). To meet this objective, the roles, responsibilities, processes, and the interactions between these roles are defined. The description of these roles and the respective responsibilities are presented in Section 4.1, and the processes and their interactions are detailed in Section 4.3. Figure 1 presents an architecture overview containing a summary of the responsibilities of each role and the simplified flow of interactions between them.

### 4.1. Roles

The formal definition of the roles that comprise this architecture is aimed to group parts with similar characteristics, to delimit the scope of the attributions and, finally, to enable the determination of responsibilities. As a result, the following roles were defined: the client (Section 4.1.1), the cloud storage service (CSS) (Section 4.1.2), the integrity check service (ICS) (Section 4.1.3), and a blockchain network (BN) (Section 4.1.4).

#### 4.1.1. Client

The client is the role that concentrates the actions defined in the architecture whose execution occurs in the infrastructure under the responsibility of the owners of files to be stored in the CSPs. The architecture defines that the client role actively exercise communication with other roles, making requests and obtaining responses directly from the services they provide. However, all interactions arising from other roles with the client are asynchronously carried out through the BN.

The main responsibilities of the client role in this architecture are:

- to encrypt the file to be stored in the cloud;
- to generate the information necessary to verify the integrity of the copies of the file during the storage period;
- to prepare and insert an instance of CFSMC with file information for each stored copy in the BN;
- to select the CSS and submit copies of the file for storage;
- to generate challenges for audit purposes when requested by the CSS;
- to hire/renew the ICS responsible for monitoring the integrity of the file copy stored in each CSS, sending the necessary information for this service execution in the contracted period.

#### 4.1.2. Cloud Storage Service

The CSS is the role that represents providers that offer a file storage service in computational clouds to a third-party (client). Under this architecture, the CSS is a service provided on demand and whose functionalities offered to the client are permanently available.

The main responsibilities of the CSS role in this architecture are:

- to receive the requisition for storage and the client’s file contents;
- to check the integrity of the received file;
- to audit the compatibility of the contents of the received file with the integrity verification information generated by the client;
- to record the acceptance of both the file storage request and the respective CFSMC;
- to reply to challenges generated by the ICS to verify the integrity of the stored files;
- to allow, at any time, the download of a copy of a stored file exclusively to the client who submitted it.

#### 4.1.3. Integrity Check Service

The ICS is the role that represents the CSPs that offer a periodic integrity-checking service of files stored in CSSs to third parties (clients) using the challenge–response method. Although the client can perform the integrity verification of its files, we strongly recommend contracting a CSP for this purpose to avoid being burdened with the infrastructure and other costs necessary to keep this service permanently active. Among the characteristics of the ICS role in this architecture, we highlight the absence of a trust relationship, i.e., from the client’s point of view, the CSP hired for this role is unreliable.

The main responsibilities of the ICS role in this architecture are:

- to provide functionality that allows the client to contract their services directly and autonomously;
- after being hired, to receive information to check the integrity of the file stored in the CSS from the client and store it;
- to generate daily challenges to verify the integrity of files stored in each monitored CSS, according to the trust level assigned to it;
- to register challenges in the BN using the linked CFSMC instance of each checked file;
- to check daily for the existence and validity of pending challenges;
- to check daily the results obtained by validating the responses received to the challenges;
- to immediately inform the client whenever the ICS identifies a breach of integrity or failure in the CSS.

#### 4.1.4. Blockchain Network

The BN is the role that represents the implementation of blockchain technology, the network formed by the nodes that carry out transactions with each other using this implementation, and the smart contracts registered in it and available to all the nodes of this network. The responsibilities assigned in the architecture for BN are implemented and available to other roles through two smart contracts called the Storage Service Trust Management Contract (SSTMC) (Section 4.2.1) and the Cloud File Storage and Monitoring Contract (CFSMC) (Section 4.2.2).

Depending on the technologies adopted, the BN is characterized by enabling the execution of transactions in a decentralized manner, not subject to interference and, mainly, without requiring a previous relationship of trust between the roles. BN also maintains a record of stored data, actions taken, and results obtained, which is public, auditable, non-editable, and resistant to attacks.

The main responsibilities of the BN role in this architecture are:

- to store one or more instances of SSTMCs;
- to store an instance of CFSMC for each file stored by a client on a CSS;
- to maintain a public record of CSPs interested in providing services as a CSS or ICS;
- to store both the storage contract data and information in each CFSMC instance to validate the answers to the integrity verification challenges;
- to receive, store, and make available to clients the challenge requests for auditing generated by CSSs;
- to receive, store, and make available to CSSs the challenges of verifying the integrity of the files stored therein;
- to receive and validate the responses to the challenges, storing them together with the result of the validation;
- to calculate and store a trust value for each CSS from the results of the challenges generated by all ICS providers, and share the results with all other roles.

### 4.2. Smart Contracts

Smart contracts are adopted as a technological solution to implement the responsibilities attributed by the architecture on the BN to ensure characteristics such as transparency, independence, and predictability. To allow for some level of flexibility in storage contracts, but also to guarantee the possibility of sharing the behavioral history of CSSs to manage trust in these services, the architecture divides these responsibilities into two smart contracts.

This division is necessary because the terms for contracting the storage service depend exclusively on the agreement between the client and the CSS involved. Therefore, the rules adopted in a contract for shared management of the trust in storage services must reach an agreement between all interested parties, that is, who will contribute what information, who will use the results, and who will be evaluated.

Given the above, in Section 4.2.1, we described the SSTMC, a smart contract designed to satisfy the responsibilities related to the management of the trust attributed to CSSs. In Section 4.2.2, we present the CFSMC, a smart contract designed to meet the responsibilities related to both file storage and monitoring.

#### 4.2.1. Storage Service Trust Management Contract

The Storage Service Trust Management Contract (SSTMC) is a smart contract designed to independently and securely carry out the necessary actions to provide a shared mechanism for classifying CSSs according to their behavior. The classification mechanism implements computational trust techniques, through which the trust value attributed to CSS (TV/CSS) is calculated and maintained. This value, in turn, expresses the expectation, based on experience, of the respective service faithfully complying with the storage contract throughout the stipulated period.

In addition to the responsibilities directly related to trust management, the SSTMC is responsible for receiving from the CSSs and registering the acceptance of a CFSMC instance. This acceptance serves as proof that the CSS has agreed to the referred storage contract terms. In addition, it allows the SSTMC to prevent a CFSMC previously unapproved by the CSS from interfering with the trust management.

Another responsibility of the SSTMC is to manage the CSPs that offer their services as a CSS or ICS. For this, the SSTMC provides public functionalities so that CSPs can self-register and self-remove. This register enables clients to consult CSPs available for each type of service. The self-registration also proves that the CSPs agreed to the rules defined in the SSTMC instance in which they self-registered.

**Figure 2:**
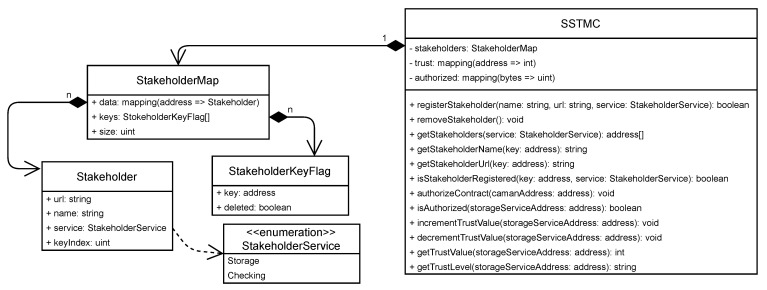
Class diagram of the SSTMC.

To fulfill these responsibilities, each SSTMC instance stores three sets of information in the BN. The first set contains the list with the data of the CSPs that performed the self-registration to make their services available (attribute “stakeholders”). The second set contains the list with the CSSs and the respective TV/CSSs (attribute “trust”). Finally, the third and last set contains the list with the accepted (authorized) CFSMC instances linked to the CSSs that accepted them (attribute “authorized”). Figure 2 shows the diagram of the class SSTMC with the signature of its methods and attributes.

The responsibilities assigned to the SSTMC are divided into functionalities (methods) classified into three groups. The first group contains the functionalities related to the management of the registered CSPs. The functionalities destined to the management of the authorizations of the CFSMC instances compose the second group. The third group includes the functionalities related to trust management. Table 1 presents the descriptions of the function of each method of SSTMC and the roles that can execute them.

The formulas adopted in the calculation processes to increase or decrease the TV/CSS, executed by the methods incrementTrustValue and decrementTrustValue, respectively, are described in Section 4.6.4. As for the conversion of TV/CSS to the trust level attributed to CSS (TL/CSS), the SSTMC uses the trust level classification model shown in Table 2 [11], which is an adaptation of the classification model proposed in Pinheiro et al. [10]. The TL/CSS and the other information in Table 2 are used in the process of generating and submitting challenges (Section 4.6.1).

#### 4.2.2. Cloud File Storage and Monitoring Contract

The Cloud File Storage and Monitoring Contract (CFSMC) is a smart contract designed to allow the monitoring of the integrity of files stored in CSSs in a transparent, publicly auditable manner. Furthermore, the CFSMC ensures that the monitoring result is free from both the third and interested parties’ interferences.

**Figure 3:**
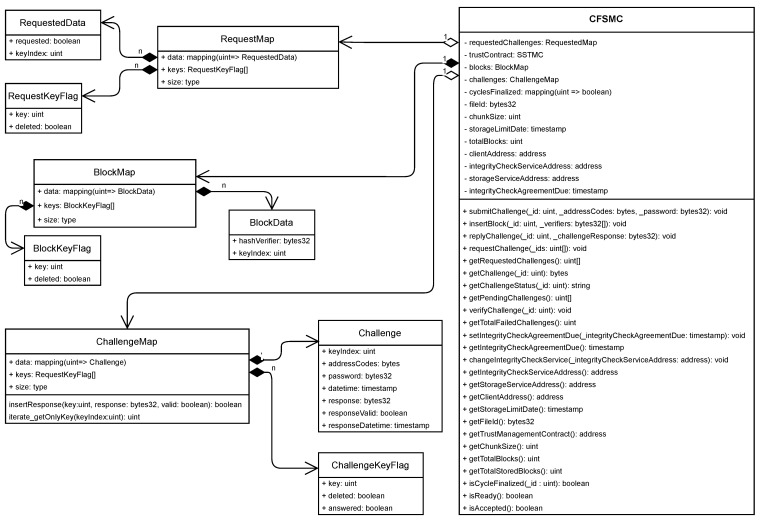
Class diagram of the CFSMC.

The responsibilities assigned to the CFSMC are: to store information on the contract and to validate responses to integrity verification challenges; to receive and store information on the contract signed with the ICS for integrity verification; to receive and store challenge requests; to receive and store challenges; and to receive challenge responses, validating them, and storing both the responses and the validation results. Figure 3 shows the CFSMC class diagram.

Each CFSMC instance, to meet its responsibilities, stores two groups of data in the BN. In the first group, each attribute stores a single value with information on the characteristics of the storage contract. In the second group, each attribute stores a set of information related to actions carried out to check the integrity of the stored file. Table 3 presents the description of the attributes present in the CFSMC.

The responsibilities assigned to the CFSMC are divided into functionalities (methods) that are classified into three groups. The first group contains functionalities that allow consulting file information and other characteristics of the storage contract. The second group gathers the functionalities related to the hiring of the ICS responsible for monitoring file integrity for a specified period. Finally, the third group comprises the functionalities associated with the reception, consultation, and validation of challenges generated to verify the integrity of the stored file. Table 4 describes the function of each CFSMC method and the roles that can execute them.

### 4.3. Architecture Processes

The processes defined in the architecture are distributed in three phases. This distribution occurs according to the moment, the role that started its execution, and the dependence between them. The first phase, called the preparation phase (Section 4.4), describes the stages of the preparation of the BN used to make it suitable for the execution of the other phases, with emphasis on the insertion of an instance of the SSTMC in the BN.

The second phase, called the storage phase (Section 4.5), details the processes carried out by the client. The main processes in this phase are the preparation of the file (encryption), the generation of the information to monitor the integrity of file during the storage period, and the submission of the file for storage in the CSS. For each file copy stored in the cloud, the storage phase describes the actions performed for the preparation and insertion in the BN of the respective instance of the CFSMC.

Finally, the third phase, named the integrity verification phase (Section 4.6), presents the processes responsible for monitoring the integrity of files stored in the cloud. Among these processes, we highlight challenge generation by the ICSs, response production by the CSSs, and the response validation by the CFSMC instances.

### 4.4. Preparation Phase

**Figure 4:**
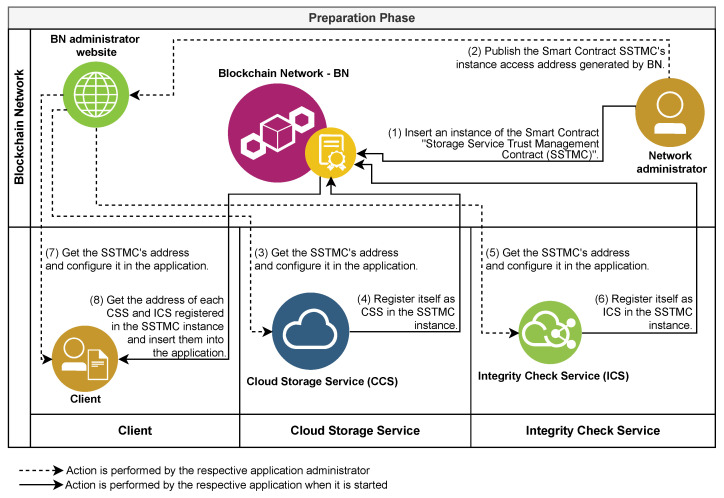
Preparation phase overview.

For the execution of the processes of the proposed architecture, we developed a reference implementation composed of three applications, each designed to meet the responsibilities assigned to one of the following roles: client, CSS, and ICS. These applications trigger the processes under the responsibility of the BN role through requests to instances of the SSTMC or CFSMC. To enable execution of the processes related to the storage and monitoring of files in the cloud, at least one instance of the SSTMC must be available in the BN and configured in the applications. Figure 4 presents an overview of the preparation phase.

The architecture preparation process begins with the BN administrator inserting an instance of the SSTMC into the BN (Step 1 in Figure 4). Each smart contract, when inserted into the BN, receives a unique access address, which is the only way to execute its functionality. For this reason, the BN administrator makes public the address of the newly inserted SSTMC instance so that interested CSPs can offer their services (Step 2 in Figure 4).

The administrators of the CSPs (CSSs or ICSs) must choose the SSTMC instance, obtain its address from the website of the BN administrator, and configure the respective applications with the SSTMC address obtained previously (Steps 3 and 5 in Figure 4). Likewise, the administrators of each client application must choose and register the respective addresses of the SSTMC instances that they wish to use (Step 7 in Figure 4).

Subsequently, at least two CSPs must offer their services through the SSTMC instance inserted in the BN: one as a CSS and the other as a ICS. To this end, the applications CSS and ICS should, on the first start-up after being configured with the address of the chosen SSTMC instance, perform self-registration in the referred contract (Steps 4 and 6 in Figure 4). The client application, in turn, obtains the list of registered CSSs and ICSs from the chosen SSTMC instance (Step 8 in Figure 4).

### 4.5. Storage Phase

**Figure 5:**
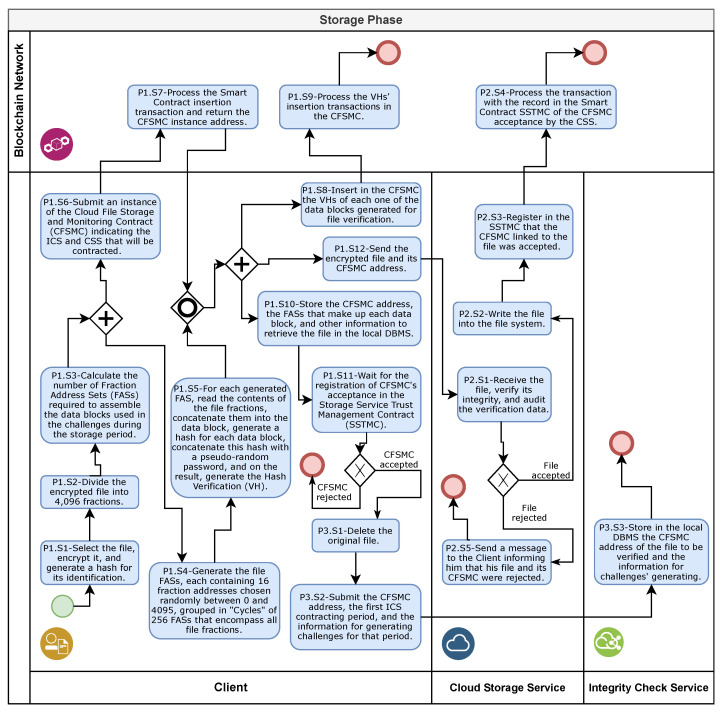
Storage phase overview.

The storage phase begins with the client with the process of selecting, preparing, and submitting copies of the file for storage in the cloud (Section 4.5.1). Then, once the file is received, the CSS executes the file storage request audit process (Section 4.5.2). Finally, after the CSS accepts the storage request, the client performs the hiring process of the ICS responsible for monitoring the file integrity (Section 4.5.3). Figure 5 provides a storage phase overview with the responsibility indications of each role.

#### 4.5.1. Selection, Preparation, and Submission of Files for Storage in the Cloud

The execution of the processes of selection, preparation, and submission of files for storage in the cloud (P1 in Figure 5) comprises 11 stages. In the first stage (P1.S1 in Figure 5), the client user makes the following selections: (i) the file whose copies will be stored in the cloud; (ii) the storage time (number of years); (iii) the SSTMC instance; (iv) the initial ICS; (v) one or more CSSs; (vi) the cryptographic key used in the encryption of the file; (vii) a unique numerical value used to ensure arbitrariness called the seed of randomness.

Still in the first stage, the selected file is then encrypted using a symmetric cryptographic algorithm and the cryptographic key (password). From the encrypted file, the client generates a hash that will serve as the file identifier. The CSS, in turn, will use this file identifier to validate the file’s integrity.

Aiming to permit the periodic checking of the integrity of the file stored in the cloud, the client assembles data blocks formed by concatenating the content of 16 file fractions chosen randomly. The collection containing the 16 integer values from 0 to 4095 (addresses) that identify each fraction content in the data block is called the fraction address set (FAS). Each address, after multiplied by the fraction size, indicates the start position of the respective fraction content.

For this, in the second stage (P1.S2 in Figure 5), based on the total size of the selected file, the client calculates the file fraction size so that the referred file can be divided into 4096 equal size fractions. Next, in the third stage (P1.S3 in Figure 5), according to file storage time and the lowest trust level defined in Table 2, the client computes the number of FASs and data blocks needed to validate the file integrity during the entire storage period without reusing the same data block.

Then, the fourth stage begins (P1.S4 in Figure 5) with the generation of the FASs according to the result calculated in the third stage. For this, the client uses the seed of randomness to arbitrarily choose 16 numbers without repetition, between 0 and 4095. Each address only can be selected again after all other 4095 addresses have been chosen. The set containing the 256 FASs and all 4096 fraction addresses of the file is called a cycle. To ensure that every FAS used in the generation of data blocks belongs to a cycle, the result of calculating the number of FASs/data blocks is always rounded to the next multiple of 256.

In the fifth stage (P1.S5 in Figure 5), the client creates a data block for each generated FAS, reading and concatenating the 16 fraction contents from the encrypted file. From each data block content, the client generates a hash, concatenates it with an exclusive password called the challenge password (CP) and from the resulting content creates a new hash named the verification hash (VH). The CP, in turn, is a hash generated from the cryptographic key and an arbitrary integer value generated by a pseudo-random algorithm, which uses the seed of randomness to guarantee its randomness.

For each chosen CSS, in the sixth stage (P1.S6 in Figure 5), the client submits a new instance of the CFSMC to the BN. To increase the overall process efficiency, this stage is started in parallel with the fourth stage. When created, each CFSMC instance receives and stores the following parameters: the file identification hash; the file fraction size; the deadline for storage; the total number of data blocks generated by the client; the address that identifies the CSS; the address that identifies the chosen ICS; the address of the adopted SSTMC instance.

The BN treats the receipt of a smart contract as a transaction that will only be persisted and made available on the blockchain after a mining node inserts it into a block in the chain and obtains the consensus of other nodes participating in the BN. The seventh stage (P1.E7 in Figure 5) is the processing of the insertion transaction of one or more CFSMC instances within the scope of the BN and the generation of the access address for each of these instances.

After waiting for the completion of the fifth and seventh stages, the Client performs the eighth stage (P1.S8 in Figure 5) with the storage of the generated VHs in the respective CFSMC instances inserted in the BN. To improve performance, the client groups 64 or more VHs to insert them into a BN in a single submission, where this number depends on the maximum limit of bytes that can be processed in a single transaction, a condition that varies according to the characteristics of each BN. The ninth stage (P1.S9 in Figure 5) represents the processing in the BN of the transactions generated by the requests for the insertion of the VHs into the CFSMC instances.

Parallel to the beginning of the eighth stage, the client executes the 10th stage (P1.S10 in Figure 5), saving the information on the file stored in the CSS in its local DBMS for monitoring and future recovery. From this information, the following are included: the file name and place of origin; the address of the respective CFSMC instance in the BN; the cryptographic key; the file verification table (FVT). The FVT contains a record for each data block generated, comprising the FAS that gave rise to it, the CP used in the VH generation, and an integer that identifies the data block and links it to the VH stored in the CFSMC. In the 11th stage (P1.S11 in Figure 5), the client monitors the SSTMC instance to identify the record that confirms whether the CSS has accepted the file storage request and the respective CFSMC instance.

The client initiates and executes the 12th stage (P1.S12 in Figure 5) in parallel with the 8th, 9th, 10th, and 11th stages. In this stage, the client performs the submission of the file copy storage request for each CSS. Attached to each storage request, the client sends the address of the respective CFSMC instance and the encrypted copy of the file to the CSS. Upon receiving the referred storage request and the file content, the CSS initiates the process of auditing and accepting the storage request for the file according to the description in Section 4.5.2.

#### 4.5.2. File Storage Request Audit and Acceptance

The execution of the file storage request audit and acceptance process comprises five stages (P2 in Figure 5). The first stage begins with the CSS receiving both the file content and the respective CFSMC instance address (P2.S1 in Figure 5). After confirming that the smart contract stored in the BN at the received address is an instance of the standard implementation of the CFSMC, the CSS obtains the file identification hash from the CFSMC. Then, to verify the received file integrity, the CSS generates a hash of its content and compares it with the referred identification hash. If the compared hashes are different, or if the received address does not point to an instance of CFSMC, the CSS rejects the file.

**Figure 6:**
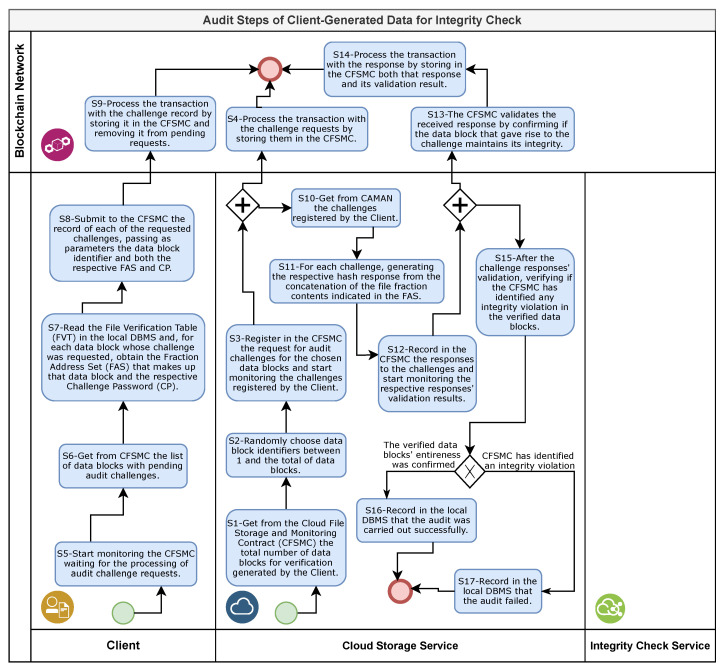
Audit steps of the data for integrity verification.

If the received file is complete, the CSS performs an audit to verify whether the data were generated by the client to verify the file integrity during its storage period are compatible with the received file. This audit consists of 17 steps, as shown in Figure 6.

The first step of the audit (S1 in Figure 6) begins with the CSS obtaining the total number of data blocks generated by the client from the CFSMC. Then, in the second step (S2 in Figure 6), using a pseudo-random algorithm, the CSS performs a draw to choose which of the client-generated data blocks will be audited.

In the third step of the audit (S3 in Figure 6), the CSS submits the challenge requests to the client through the CFSMC. Each challenge request receives as a parameter a list with the identifiers of the data blocks, the contents of which the CSS has chosen to verify the integrity. The total number of challenges requested is equal to one-tenth of the number of verification cycles generated by the client. As the client generates 256 data blocks in each cycle, for every 2560 data blocks generated by the client, the CSS will require the client to submit a challenge for auditing purposes.

In the fourth phase of the audit, the BN node, when receiving the submission with the challenge requests (S4 in Figure 6), inserts a new transaction in the BN and waits for a mining node to include it in a new block in the blockchain and replicate it to the other participating nodes. Upon completion of the transaction processing, information on the requested challenges will be available to the client.

If the file submitted for storage is not accepted by the CSS or receives a message indicating its rejection, the client, in turn, performs the fifth stage of the audit (S5 in Figure 6), in which the client monitors the CFSMC until the CSS records the audit challenge requests. In the sixth step (S6 in Figure 6), the client obtains a list with the identifiers of the data blocks whose challenges were requested from the CFSMC. Then, in the seventh step (S7 in Figure 6), the client reads the file verification table (FVT) in the local DBMS and, for each data block identifier of the requested challenges, obtains both the respective fraction address set (FAS) and challenge password (CP).

Then, the eighth step of the audit (S8 in Figure 6) begins when the client submits the requested challenges for registration in the CFSMC, providing as parameters the data block identifier, the FAS that gave rise to this data block, and the CP used in the generation of the respective verification hash (VH). The ninth step (S9 in Figure 6) represents the processing of transactions generated in the BN by the client node when registering challenges. In this step, the CFSMC stores the received challenges in the blockchain and automatically removes the respective requests from the pending challenge requisition list.

The CSS, in turn, while there are pending requests, periodically monitors the challenges registered in the CFSMC. In the 10th audit step (S10 in Figure 6), the CSS obtains the FAS of each pending challenge from the CFSMC. Then, in the 11th audit step (S11 in Figure 6), the CSS reads the contents of the fractions indicated by the FAS from the received file and concatenates these contents, forming the data block. From each of these data blocks, the CSS generates a response hash to the respective challenge. Next, in the 12th audit step (S12 in Figure 6), the CSS answers the challenges registered by the client by submitting the respective response hashes to the CFSMC.

Upon receiving these responses, the CFSMC initiates the 13th audit step (S13 in Figure 6), in which it validates the answer to each challenge and removes them from the pending challenges list. For this, the CFSMC firstly concatenates the response hash with the CP registered in the respective challenge, builds a hash from this content, and compares this generated hash with the VH of the respective data block stored in the CFSMC. The 14th step of the audit (S14 in Figure 6) represents the process of reaching a consensus from the BN to the transaction generated by the response submission. This consensus results in the storage and distribution of a new block in the blockchain to all BN nodes containing both the hash response and its validation result.

In sequence, the CSS performs the 15th step of the audit (S15 in Figure 6), verifying in the CFSMC if the validation of the responses to the challenges has confirmed that all data blocks verified have maintained their integrity. In this case, in the 16th step of the audit (S16 in Figure 6), the CSS records in the local DBMS that the audit confirmed that the data integrity verification generated by the client is compatible with the file received. Otherwise, the CSS performs the 17th step of the audit (S17 in Figure 6), recording that the audit failed in the local DBMS and that the received file is incompatible with the client-generated verification data.

If the audit was successful, the CSS starts the second stage of the process (P2.S2 in Figure 5), moving the received client file from the temporary storage area to a definitive area. The information on file, such as the CFSMC instance address and the storage location, are saved in the local DBMS. Then, in the third stage (P2.S3 in Figure 5), the CSS records the acceptance of both the file and the CFSMC instance linked to it in the SSTMC instance.

The fourth stage (P2.S4 in Figure 5) represents the processing in the BN of the transaction resulting from the registration of the CFSMC acceptance by the CSS. In this processing, the SSTMC checks whether the address registered by the client in the referred CFSMC as the smart contract responsible for managing trust in the CSS is equal to its access address. The SSTMC also checks whether the request source address is a registered CSS, is active, and is the same address registered by the CSS in the CFSMC. Upon identifying the referred acceptance, the client starts the process of hiring the service for monitoring file integrity (P3 in Figure 5) described in Section 4.5.3.

If the audit failed or the received file integrity was not confirmed, the CSS ignores the second, third, and fourth stages and performs the fifth stage (P2.S5 in Figure 5). In this stage, the CSS deletes the received file and sends a message to the client stating the rejection of the storage request.

#### 4.5.3. Hiring Service for Monitoring File Integrity

After identifying the acceptance of the storage request by the CSS, the client begins the process of hiring the monitoring file integrity service (P3 in Figure 5). In the first stage (P3.S1 in Figure 5), the client registers the CSS acceptance of the file storage request in its local DBMS. Following confirmation that the respective CSSs accepted the storage request of all file copies, the client deletes the original file.

Then, the client performs the second stage of this process (P3.S2 in Figure 5), calculating and submitting the necessary information for the challenge generation to the ICS. Based on the lowest trust level defined in Table 2, the client calculates the number of verification cycles necessary to generate challenges during the contracted time, whose renewal periodicity must be previously defined by the client. Next, the client extracts (reads and deletes) the information on the data blocks necessary from the FVT in the local DBMS and submits them to the ICS together with the CFSMC instance address and the monitoring contract end date. The data block identifier and both the respective FAS and CP compose the referred data block information for each predicted challenge.

At the end of the storage phase, in the third stage of this process (P3.S3 in Figure 5), the ICS stores the address of the CFSMC instance linked to the monitoring object file, the deadline of the monitoring contract, and the information to generate the challenges in the local DBMS. At the end of the monitoring contract period, after analysis and confirmation of the ICS correctness for the referred period, the client renews the hiring by re-executing the second and third stages of this process.

### 4.6. Integrity Verification Phase

**Figure 7:**
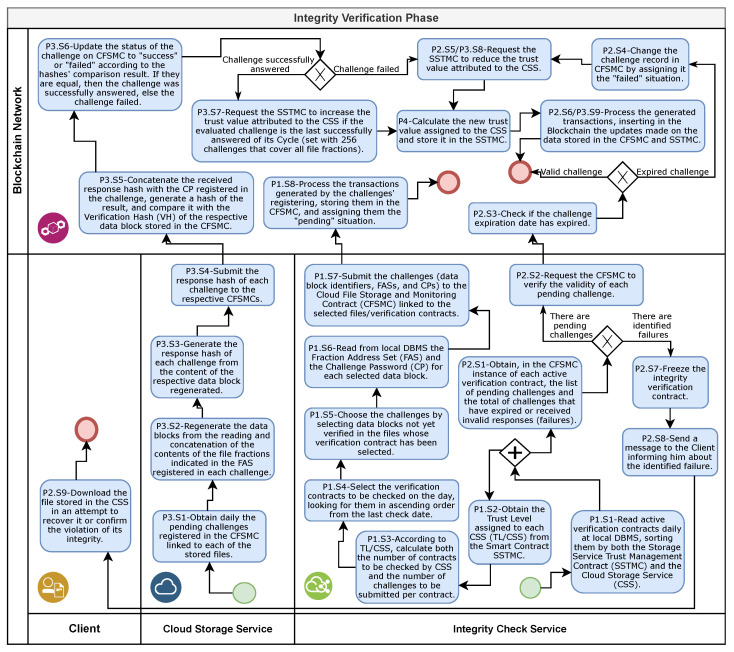
Integrity verification phase overview.

The integrity verification phase describes the processes conducted by the ICSs and CSSs to periodically confirm the integrity maintenance of files stored in the cloud. To this end, the ICSs generate daily challenges to the CSSs and register them in the CFSMC linked to the monitored files. In parallel, each CSS monitors the pending challenges registered in the CFSMC instances of the files that it stores, generates the respective responses, and records them in the original CFSMC. Finally, each CFSMC validates and stores the received responses and, when necessary, triggers the SSTMC to update the trust attributed to the respective CSS. Figure 7 presents an overview of the integrity verification phase.

The execution of the integrity verification phase is divided into four processes. Section 4.6.1 presents the challenge generation and submission process (P1 in Figure 7). The previous challenge verification process (P2 in Figure 7) is described in Section 4.6.2. Section 4.6.3 describes the challenge response generation, submission, and verification process (P3 in Figure 7). Finally, the trust value calculation process (P4 in Figure 7) is presented in Section 4.6.4.

#### 4.6.1. Challenge Generation and Submission

The execution of the challenge generation and submission process (P1 in Figure 7) comprises eight stages. The first stage (P1.E1 in Figure 7) starts in the ICS with the reading of the active integrity verification contracts in the local DBMS. The ICS executes this reading once a day, ordering the read contracts according to both the SSTMC and CSS instances.

Then, in the second stage (P1.S2 in Figure 7), the ICS obtains the updated value of trust level assigned to CSS (TL/CSS) from the SSTMC for each CSS present in at least one active verification contract. Parallel to the execution of this stage, the ICS starts the previous challenge verification process (Section 4.6.2).

Starting the third stage (P1.S3 in Figure 7), the ICS sums the number of files with active verification contracts by CSS. Next, for each CSS, using the percentages defined in Table 2 and according to TL/CSS, the ICS calculates how many files will have their integrity checked for that day and how many challenges to generate for each file. Then, the ICS performs the fourth stage of this process (P1.S4 in Figure 7), selecting the verification contracts whose monitored files in each CSS will receive challenges on that day following the ascending date and time order of the last submitted challenge.

In the fifth stage (P1.S5 in Figure 7), for each verification contract selected, the ICS chooses which data blocks to check on that day from amongst the data blocks not yet used in previous challenges, following the ascending order of the data block identifier belonging to the last-used verification cycle, or selects a new cycle if the last has already been finalized. Next, in the sixth stage (P1.S6 in Figure 7), the ICS reads the FAS and CP in the local DBMS for each chosen data block in the verification contracts selected to receive challenges. Then, the ICS executes the seventh stage (P1.S7 of Figure 7), submitting the challenges to the respective CFSMC using the identifier, FAS, and CP of one of the chosen data blocks to generate each one.

To complete this process, the BN performs the eighth and final stage of this process (P1.S8 in Figure 7), which represents the processing of transactions generated by the CFSMC when receiving the challenges submitted by the ICS, assigning to them the situation “pending”, and storing them. After the processing the referred transactions, with the insertion of one or more new blocks in the chain, followed by the distribution and obtaining of a consensus with the other BN nodes, the registered challenges will then be available for the generation, submission, and verification of the responses to the challenges process (Section 4.6.3).

#### 4.6.2. Verification of Previous Challenges

The execution of the verification process of the previous challenges (P2 in Figure 7) comprises nine stages. In the first stage (P2.S1 in Figure 7), for each active verification contract, from the respective CFSMC, the ICS obtains a list of all challenges registered with the situation of “pending” and the total of challenges with the situation of “failed”, i.e., challenges that expired without receiving a response or whose response was considered invalid. Then, the ICS performs the second stage of this process (P2.S2 in Figure 7), requiring the CFSMC with one or more pending challenges to verify the validity of these challenges.

Upon receiving the referred request, in the third stage (P2.S3 in Figure 7), the CFSMC verifies each pending challenge and checks whether the period waiting for a response exceeded 72 h. In this case, the CFSMC executes the fourth stage (P2.S4 in Figure 7), changing the challenge situation to “expired”. Next, in the fifth stage (P2.S5 in Figure 7), the CFSMC requests the SSTMC instance to reduce the TV/CSS. The receipt by the SSTMC of the referred request starts the trust value calculation process (P4 in Figure 7) described in Section 4.6.4. After the SSTMC concludes this calculation, the BN performs the sixth stage (P2.S6 in Figure 7), which represents the processing of the transaction generated by the CFSMC to update the challenge situation, as well as the transaction generated by the SSTMC to update the TV/CSS.

In parallel with the execution of the second stage (P2.S2 in Figure 7), the ICS starts the execution of the seventh stage (P2.S7 in Figure 7). In this stage, for each CFSMC where the query to the total challenges registered with the situation “failed” resulted in a value other than zero, the ICS changes the respective file verification contract status from “active” to “frozen”. Notably, the referred contract will remain in frozen status until the client requests reactivation or definitive cancellation.

Subsequently, in the eighth stage (P2.S8 in Figure 7), the ICS sends a message (e-mail) informing each client whose file checking contract was frozen of the failure. The client, in turn, when informed of the identified fail, starts the execution of the ninth and last stage of this process (P2.S9 in Figure 7), requiring the download of the copy of the respective file from the CSS. If the download is successful, the client confirms file integrity by comparing the hash of the downloaded content with the identification hash obtained from the respective CFSMC. Once file integrity is proven, the client requests the ICS to reactivate the verification contract or, in the event file integrity violation is proven, the client requests the definitive cancellation of the contract.

#### 4.6.3. Generation, Submission and Verification of the Responses to the Challenges

The execution of the generation, submission, and verification of the responses to the challenges process (P3 in Figure 7) comprises nine stages. The ICS starts and executes the first stage (P3.E1 in Figure 7) in parallel with the first stage of the challenge generation and submission process (P1.S1 in Figure 7). In this stage, the CSS reads daily the instance address of all stored files in the CFSMC and obtains the pending challenges from each CFSMC instance.

In the second stage of this process (P3.E2 in Figure 7), for each storage contract with pending challenges, the CSS obtains the hash identifier and fraction size from the respective CFSMC instance. Next, for each pending challenge, the CSS obtains the FAS from the CFSMC, accesses the file through the hash identifier, and reads the contents of 16 file fractions according to addresses indicated in the FAS. For this, the CSS calculates the initial position of each file fraction by multiplying its address by the fraction size. Then, the CSS concatenates the read fraction contents to regenerate the data block that produced the challenge.

After completing the generation of the data blocks indicated in the challenges, the CSS performs the third stage (P3.S3 in Figure 7), generating a response hash from each regenerated data block content. Then, in the fourth stage (P3.S4 in Figure 7), for each challenge, the CSS submits the respective hash response to the challenge-origin CFSMC instance.

In the fifth stage of this process (P3.S5 in Figure 7), the CFSMC validates each received response hash. For this, the CFSMC concatenates the received hash response with the CP registered in the respective challenge, generates a hash from this concatenated content, and compares this hash with the respective stored VH. When the compared hashes are equal, the challenge is successfully answered. According to the result obtained in the validation of each response hash, in the sixth stage (P3.S6 in Figure 7), the CFSMC updates the respective challenge, changing its pending status to success or failed.

Next, if the challenge is successfully answered, the CFSMC performs the seventh stage (P3.S7 in Figure 7), checking whether the referred challenge is the last in the verification cycle to which it belongs and whether all other 255 challenges in this cycle have also been successfully answered. In this case, the CFSMC requests the SSTMC instance to increase the TV/CSS. Otherwise, if the challenge fails, the CFSMC performs the eighth stage (P3.S8 in Figure 7), requesting the SSTMC instance to reduce the TV/CSS.

If the TV/CSS update is requested, the SSTMC performs the trust value calculation process (P4 in Figure 7) described in Section 4.6.4. Then, the CFSMC executes the ninth and final stage of this process (P3.S9 in Figure 7), which represents the processing of the transaction generated by the CFSMC to store the challenge response and the challenge status update, as well as the transaction generated by the SSTMC to update the TV/CSS when requested.

#### 4.6.4. Trust Value Calculation

The trust value calculation process (P4 in Figure 7) uses the trust calculation model proposed by Pinheiro et al. [10] and adapted by [11] to allow its implementation in smart contracts and to accelerate the penalization of services that present recurrent failures with the progression of their classification through the various levels of distrust. Conversely, the trust model should also ensure that the CSPs, which maintain their history free from the integrity violation records and strictly comply with the deadlines for generating responses to challenges, are gradually reclassified from the lowest to the highest trust level.

Upon receiving a request to update the trust value attributed to a CSS, the SSTMC starts the calculation process, obtaining the current trust value assigned to the referred CSS. Next, according to the type of request, the SSTMC executes Algorithm 1, to reduce the TV/CSS; or Algorithm 2, to increase the TV/CSS. In both algorithms, trustValue represents the current TV/CSS and newTrustValue receives the updated TV/CSS.
**Algorithm 1.** Reduction in the trust value. **if** 
trustValue>0
 **then**  newTrustValue←0 **else**   **if** trustValue=0
 **then**$\;\;\;newTrustValue\leftarrow -1.5\times 10^{19}$   **else**    **if** $trustValue\geq -5\times 10^{19}$
 **then**    newTrustValue←trustValue−(trustValue×−0.15)    **else**$\;\;\;\;newTrustValue\leftarrow trustValue-(\left(-1\times 10^{20}-trustValue\right)\times -0.025)$**    end if****   end if** **end if**


**Algorithm 2.** Increase in the trust value. **if**
 trustValue<0
 **then**
$\;\;newTrustValue\leftarrow trustValue+(\left(-1\times 10^{20}-trustValue\right)\times -0.025)$
 **else**   **if** trustValue=0
 **then**
$\;\;\;newTrustValue\leftarrow 1.5\times 10^{19}$
   **else**    **if** $trustValue\geq 5\times 10^{19}$
 **then**
    newTrustValue←trustValue+(trustValue×0.025)
    **else**
$\;\;\;\;newTrustValue\leftarrow trustValue+(\left(1\times 10^{20}-trustValue\right)\times 0.005)$
    **end if**   **end if** **end if**

## 5. Architecture Validation

This section presents the validation of the applicability, effectiveness, and efficiency of the architecture proposed in [11] when applied to large files and for a long storage period. For this validation, we used nine files with sizes varying between 50 MB and 10 GB, and with an estimated storage time in the cloud ranging between 1 and 25 years.

### 5.1. Infrastructure

To perform this validation, we used a laboratory with configurations similar to that used in the tests presented in [11], to allow a better comparison of the obtained results. However, we increased the storage capacity and standardized the amount of RAM used due to the number, size, and estimated cloud-storage time of the files used. This laboratory had six equal virtual machines (VMs) with Intel(R) Xeon(R) CPU E5-2660 v3 @ 2.60 GHz processors, 64 bits, one core, 300 GB HD, a Linux Operating System, kernel version 4.15.0-55-generic, distribution Ubuntu 18.04.4 LTS, and 12 GB of RAM.

As in [11], we installed the application that implements the CSS role features in three VMs, and in the other three VMs, we installed the application that implements the ICS role features. To execute the tests, due to the limitation of the available laboratory, one of the VMs destined for the role of ICS had its processing capacity shared with the application destined to the client role. We used Postgresql software as the DBMS for all applications, and for the CSS and ICS applications, we used Glassfish Server Open software as the application server.

In each of these VMs, in addition to the specific applications for each role, we installed a node of the private Ethereum BN created exclusively to perform this validation. We configured this network to use proof-of-authority as a consensus algorithm, five-second intervals between the creation of each block, $7.5\times 10^{6}$ as the ideal sum of the gas consumption of the transactions inserted in the same block, $1.0\times 10^{7}$ as the maximum consumption, and 100 *wei* as the price of each gas unit.

### 5.2. File Submission

To start the validation process, we inserted a single instance of the SSTMC for trust management in the BN, for which we configured three CSSs and three ICSs to self-register. Using the client application, we submitted a copy of each of the nine files available for storage in each of the three accessible CSSs, with different storage times, selecting the first ICS as the verification service for the 27 copies. Subsequently, we repeated this process of submitting files for storage two times, choosing the second and third ICS in each, totaling 81 stored files. Table 5 summarizes the average time spent preparing the files according to the file size and the expected storage time.

### 5.3. Effectiveness and Efficiency Validation

To validate the architecture’s effectiveness in monitoring the integrity of large files stored in the cloud for long periods, we used the same tool applied in the validations presented in [11]. This tool allowed simulating the passing of a day, forcing the ICS to perform its daily process of checking the results of previous challenges and generating new challenges every *n* minutes. In addition, the tool recorded the results obtained monitoring each file and the variations in the trust level assigned to each CSS. Due to the size and quantity of files used in this validation, the interval time used to simulate the passage of a day was 3 min.

**Figure 8:**
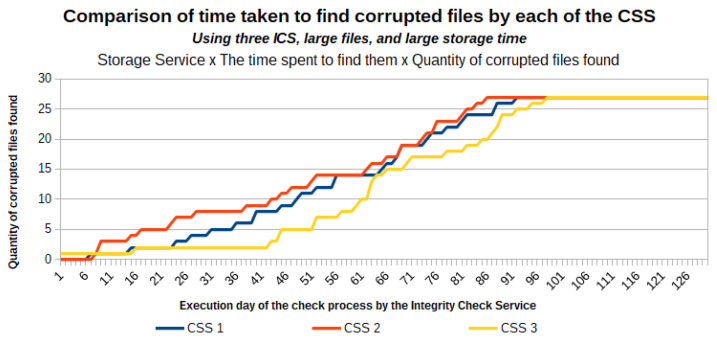
Comparison of the periods required to identify corrupted files in each of the CSSs.

To determine if the architecture is effective in identifying the simplest flaws, both in small and large files, we replaced the content of one randomly chosen byte in each of the file copies stored in the three CSSs. After completing the validation environment preparation, we started the integrity checking process of the stored files using the simulation tool. Next, over fewer than 100 days of execution, the faults, previously inserted in the 81 files, were already identified. Table 6 shows the day of execution when the architecture found each implanted failure, and a comparative graph of the time spent to find the flaws in the files stored in each CSS is shown in Figure 8.

**Figure 9:**
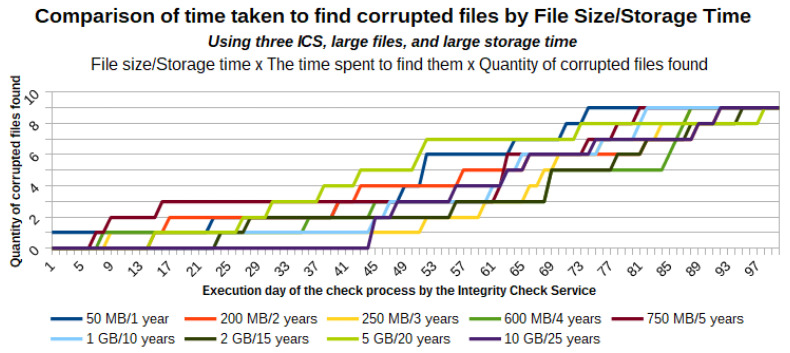
Comparison of the periods required to identify corrupted files by file size and storage time.

The results obtained in this validation process confirmed the effectiveness of the architecture, regardless of file size and expected storage time. After grouping the validation results according to file size and respective storage time, we observed that the average time to identify a failure in a file varied between 46.8 and 66.8 days and that this variation in the average time was not directly related to the increase in storage time or file size. Table 7 presents a summary of the results obtained according to file size and storage time in ascending order of average time to identify the failure in a file. Figure 9 depicts a comparative graph of when the architecture found the flaws in each file according to file size and storage time.

To conclude the analysis, we compared the results obtained in this architecture validation process with the test results presented in [11], where the predicted storage time was the same for all files (one year), the largest tested file was 1 GB, and the total number of monitored files was 54. The comparison result confirms the efficiency of the architecture regardless of quantity, size, and expected storage time of the files used because the average time to identify a failure varied only from 57.07 to 57.4 days.

**Figure 10:**
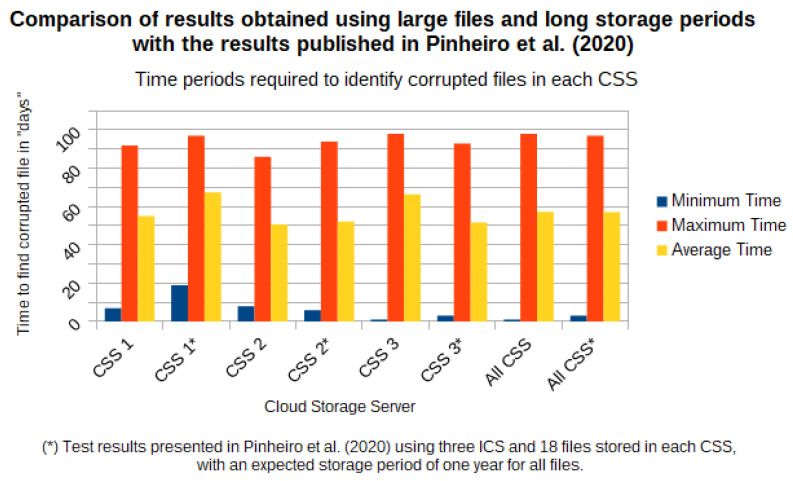
Comparison of results obtained using large files and long storage periods with the results published in Pinheiro et al. [11].

Although the performed tests increased the number of monitored files by 50%, the largest file size by 1000%, and up to 2500% in storage time, the identified time variation represented an increase of only 0.57% in the average time spent finding failures. Figure 10 compares the results obtained by each CSS in this validation to the results of the tests published in [11], and Table 8 summarizes the results obtained in this validation.

## 6. Security Analysis

From a security perspective, we defined the processes executed in the architecture proposed in this research considering that the roles client, ICS, and CSS would act according to the malicious adversarial model concept. In this model, it is assumed that the parties involved in a process are unreliable. That is, they can send invalid data, obtain sensitive information, leak information, fail to follow the steps provided, or act in collusion to harm other parties [62].

The BN role is considered trustworthy by all other roles because its responsibilities are implemented through smart contracts, a technology characterized by being difficult to be violated, as described in Section 2.3. In sequence, we describe the limitations of the research conducted in Section 6.1; in Section 6.2, we present the main attack scenarios on the proposed architecture whose sources are the client, ICS, and CSS roles. For each attack scenario, we present the defense mechanisms adopted in the architecture to prevent or reduce the probability of success of an attack.

### 6.1. Research Limitations

Smart contracts are an emerging technology for which it is assumed that there are still undiscovered vulnerabilities [63]. Once deployed in BN, smart contracts are difficult to modify, and if there are security vulnerabilities, it would be hard to prevent or contain any attacks. To minimize the risks of becoming vulnerable, we performed the encoding of smart contracts SSTMC and CFSMC following the best security practices described in [64].

As the reasons for insecurity are the limitations of the adopted technology, which may or may not be effective, we assumed that the blockchain platform and the smart contracts used in the environment are safe. We also think that an opponent would not gain control of more than 49% of the BN nodes, which means an attacker would neither be able to modify the information stored in the blockchain nor interfere with the behavior of the smart contracts during their execution.

Likewise, it is expected that at any given moment, with the evolution in current computers and the consequent growth in computational power available to users, the cryptographic mechanisms currently available would become inefficient. However, this risk is another technological limitation that was not covered within the scope of this research and, because of this, we assumed that both the cryptographic algorithms and hash sizes used are safe and will remain so throughout the period of file storage in the cloud.

### 6.2. Resistance against Attacks

**Attack 1.**

The client sends a corrupted file to the CSS.

Defense mechanism: The client generates and inserts the file hash in an instance of the CFSMC and stores it in the BN. When receiving the file together with the address of the respective CFSMC instance, the CSS generates the hash of this file and compares it with the hash stored in the respective CFSMC, rejecting the received file if the compared hashes are different.

**Attack 2.**

In the CFSMC instance linked to its file, the client stores the hashes to verify the responses to the challenges with content incompatible with the file/challenge.

Defense mechanism: After receiving the file, the CSS randomly chooses a percentage of the challenge verification hashes registered in the respective CFSMC and requires the client to submit the corresponding challenges, which are answered by the CSS and validated independently by the CFSMC. The CSS rejects the file received if the CFSMC does not consider all responses to these challenges valid.

**Attack 3.**

The ICS does not comply with the rules provided in the architecture and fails to submit the daily amount of challenges to the CSS.

Defense Mechanism: The client periodically chooses an ICS, a CSS, and an audit date, after which the client obtains instances from the CFSMC, linked to the stored files and monitored by the chosen CSS and ICS, respectively, and the total number of challenges registered for each file on the chosen date. Next, the client obtains the TL/CSS from the SSTMC on the audited date. Using the obtained TL/CSS, the client computes the number of challenges that should have been submitted to the CSS on the referred date. If the client identifies any discrepancy between the number of challenges submitted by the ICS and the amount calculated according to the TL/CSS on the audited date, the client begins the process of replacing the contracted ICS.

**Attack 4.**

The CSS creates the hash responses for all challenges previously generated by the client from information on these challenges improperly made available by the ICS, stores these responses, and deletes the stored file.

Defense mechanism: The client only sends the information to the ICS to generate challenges for the contract validity period between the ICS and the client. This period must be shorter than the period established for storing the file in the CSS. For this reason, even if the CSS receives information about the challenges in advance from the ICS and it pre-calculates the responses to the challenges of that period, the CSS would not be able to delete the client file as it would not be able to predict for what challenges the client will submit information to the ICS after the renewal of the contract between them.

**Attack 5.**

After receiving the file for storage, the CSS pre-calculates and stores the responses for all possible challenges and deletes the received file.

Defense mechanism: The Client generates the challenges before submitting the file for storage based on the concatenation of the content of the randomly chosen file fractions. For this, the client divides the file into 4096 fractions and, for each challenge, randomly chooses 16 non-repeated fractions (simple arrangement). Since the possible number of combinations of these fractions ($C_{\left(4096,16\right)}$) is approximately $6.09\times 10^{57}$ and the length of the answer for each challenge is 32 bytes, the CSS would need to store almost $1.5\times 10^{47}$ TB of data to ensure that it has all the answers to the possible challenges generated for a single file, which makes the attack impracticable because it surpasses the cost of storing the file.

**Attack 6.**

The CSS uses the information used by CFSMC to validate the responses to the challenges, which are publicly stored in the BN to identify the challenges generated by the Client, stores the responses, and deletes the stored file.

Defense mechanism: For the validation of the response generated by the CSS and registered in the CFSMC instance linked to the file, the referred smart contract needs to concatenate the CSS response with a 256 bit hash generated by the client from a pseudo-random and exclusive password for each challenge, which is only made available to the CFSMC when the ICS records the challenge. Therefore, to find only one challenge, it would be necessary, on average, to compare the response of half of the $6.09\times 10^{57}$ possible combinations of fractions that produce the challenge concatenated with half of each of the $1.15\times 10^{77}$ possible password combinations.

**Attack 7.**

The CSS repeats the challenge response that has already been answered and validated successfully to artificially improve its trust level.

Defense mechanism: The CFSMC, when receiving the answer to a challenge, verifies whether there is a recorded response for the referred challenge and, in this case, it simply disregards the answer. Additionally, the CFSMC implements a mechanism that ensures that the TV/CSS increase/decrease can be requested only once for each cycle of challenges.

**Attack 8.**

The client inserts a smart contract with a different implementation than the one established for the CFSMC in the BN, intending to simulate integrity violations and reduce the TV/CSS, consequently compromising the CSS reputation.

Defense mechanism: The SSTMC implements a routine in the methods responsible for receiving the requests to increase/decrease the TV/CSS that verifies whether the address of the smart contract from which the request originates is on the list of smart contracts previously authorized by the respective CSS, discarding the request when not found. The CSS recognizes a CFSMC through the CFSMC acceptance registration in the SSTMC instance as the final result of the storage request audit and acceptance process. In this process, among other actions, the CSS checks whether the smart contract address, received with the storage request, points to an instance of the standard implementation of the CFSMC stored in the BN. Otherwise, the CSS immediately rejects the file.

## 7. Limitations

The number of files simultaneously submitted for storage in the CSPs is limited to the BN’s capacity to process the transactions that store the CFSMC instance linked to each file and information for validating the challenges. This limitation occurs because the proposed architecture requires the client to provide the address on the BN of the CFSMC instance linked to the file before submitting its content for storage in the CSP. However, the client will only obtain the referred address after the BN processes the transaction containing the client’s request for the CFSMC instance insertion in the blockchain.

The maximum storage time with the security monitoring guaranteed by the architecture is limited to the validity period of the security provided by the encryption and hashing algorithms adopted in the architecture implementation. The current security can be broken in the future due to the discovery of vulnerabilities in the adopted cryptographic solution implementations or an exponential increase in the available computational power that will make the brute force attacks on them efficient.

Although the architecture proposed in this work predicts the possibility of being used in public BNs, as it was beyond the scope of the current research phase, the costs related to fees charged by the nodes responsible for inserting new transaction blocks in the blockchain were not analyzed. Furthermore, the possible impacts of the flow of transactions generated by the architecture on the fluctuation in the value of fees charged by the BN to process the transactions were also not evaluated.

## 8. Conclusions

The use of solutions based on cloud computing was presented as a viable alternative to reducing the costs and the complexity of managing institutions’ information technology resources. Pinheiro et al. [11] proposed a software architecture that allows the storage of files in cloud services, with guaranteed information privacy and permanent monitoring of the integrity of the files, based on technologies such as blockchain, smart contracts, and computational trust.

Among the possible applications for the proposed architecture, we highlight the storage of backups of the database of electronic document management systems, which, in most cases, are large files and, due to legal issues, need to be stored for long periods. In this sense, this article presented a validation of the effectiveness and efficiency of the referred architecture when used for the storage and monitoring of large files for long periods. The article also presented an analysis of the security of this architecture.

From the results obtained in that validation process, it was initially possible to verify that the average time spent in the steps that comprise the process of preparing the files for submission to the cloud grew in proportion to the file size and the expected storage time, as shown in Table 5. Most of this time spent is the client generating the challenge verification hashes for the content of each data block.

The changed byte detection in each of the 81 monitored files up to 10 GB in size and expected storage time of up to 25 years occurred in an average period of 57.4 days, as shown in Table 8. This result confirmed that the architecture maintained its efficiency when used with large files and longer periods of storage, and proved the architecture’s effectiveness in identifying flaws in files regardless of file size or storage time. The confirmation occurred because even with a 50% increase in the number of files, up to a 1000% increase in file size, and up to a 2500% increase in storage time, the average time to identify an adulterated file increased only 0.57% (0.33 day) compared to the results obtained in Pinheiro et al. [11].

To conclude, we presented an analysis of the main attacks expected against the functioning of the proposed architecture. For each described attack, we showed the defense mechanism that guarantees the architecture’s security against the attack, and explained how the executed actions nullify the possibility of the attack’s success.

### Future Works

For future research, we intend to propose an improved version of this architecture that implements a dynamic mechanism to define the number of fractions by file and the number of these fractions that compose each challenge, both varying according to the file size. This change aims to improve the performance of the process of preparing files larger than 10 GB while maintaining the efficiency of the adulterated file identification process.

Given the existence of several blockchain platforms with different characteristics and applications, we plan to implement and test the proposed architecture on some of these platforms, such as Hyperledger Fabric. The aim is to measure the architecture’s efficiency gain or loss in each of these platforms, identifying the advantages, disadvantages, and limitations of the architecture. For each tested platform, based on its implementation characteristics and obtained results, we hope to identify, propose, and test improvements in the architecture processes that help to increase the overall architecture efficiency.

For this research sequence, another objective is to analyze the costs and the feasibility of adopting the proposed architecture using a public BN. For this purpose, we intend to conduct a set of tests to determine the expected costs concerning the processing fees of the transactions generated by each of the process phases defined in the architecture. In addition, based on the results obtained, we intend to identify opportunities to suggest improvements in the proposed architecture to reduce transaction costs, focusing on the phases that represent the largest share of the total cost of operation.

We also propose in future work to determine the applicability of artificial intelligence (AI) techniques to allow the architecture processes to self-adapt to changes in the environment (e.g., the increase in network latency or unexpected growth in demand for a particular CSP). Among the possible improvements through the adoption of AI, we highlight the implementation of intelligent load balancing for the challenge generation process. This load balancing must consider scheduling its execution during the periods of the day with less network traffic and the least amount of pending processing processes in the BN. We also highlight the use of dynamic rules in the calculation of TL/CSS that consider, in addition to the number of challenges answered correctly, the number of files stored by each CSS, minimizing differences in the speed of the evolution of the TL/CSS between large and small providers.

## Figures and Tables

**Table 1:** Functions of SSTMC methods and the roles that can perform them.

Method	Group	Function	Execution
authorizeContract	2nd	Record acceptance of a storage contract (instance of the CFSMC)	CSS registered
decrementTrustValue	3rd	Run the calculation process to reduce the TV/CSS	CFSMC
getStakeholderName	1st	Provide the name of a CSS/ICS	Public
getStakeholders	1st	Provide a list of registered and available CSSs or ICSs for hire	Public
getStakeholderUrl	1st	Provide the URL to access the services of the CSS/ICS	Public
getTrustLevel	3rd	Provide the trust level assigned to the CSS computed from the updated TV/CSS	Public
getTrustValue	3rd	Provide the updated TV/CSS	Public
incrementTrustValue	3rd	Run the calculation process to increase TV/CSS	CFSMC
isAuthorized	2nd	Inform if the requesting CFSMC instance was accepted by the CSS	CFSMC
isStakeholderRegistered	1st	Inform if a CSS/ICS is registered and available for hire	Public
registerStakeholder	1st	Allow CSPs to perform their own registration and offer their services as a CSS or ICS	CSSs and ICSs
removeStakeholder	1st	Allow a CSP to block its own registration so that clients can no longer hire it as a CSS/ICS	CSS and ICS registered

**Table 2:** Trust level classification [11].

Trust Level	Trust Value Range	Checked per Day		
		% from Files	% of the File	DataBlocks
Very high trust	$]9\times 10^{19}$, $1\times 10^{20}[$	15%	∼0.4%	1
High trust	$]7.5\times 10^{19}$, $9\times 10^{19}]$	16%	∼0.8%	2
Medium-high trust	$]5\times 10^{19}$, $7.5\times 10^{19}]$	17%	∼1.2%	3
Low-medium trust	$]2.5\times 10^{19}$, $5\times 10^{19}]$	18%	∼1.6%	4
Low trust	[0, $2.5\times 10^{19}]$	19%	∼2.0%	5
Low distrust	$]-2.5\times 10^{19}$, 0[	20%	∼2.4%	6
Low-medium distrust	$]-5\times 10^{19}$, $-2.5\times 10^{19}]$	25%	∼3.2%	8
Medium-high distrust	$]-7.5\times 10^{19}$, $-5\times 10^{19}]$	30%	∼4.0%	10
High distrust	$]-9\times 10^{19}$, $-7.5\times 10^{19}]$	35%	∼4.8%	12
Very high distrust	$]-1\times 10^{20}$, $-9\times 10^{19}]$	50%	∼5.6%	14

**Table 3:** Description of the attributes present in the CFSMC.

Attribute	Group	Description
blocks	2nd	Associative matrix key/value containing the verification hash (VH) of each data block generated by the client to verify file integrity through its fractions
challenges	2nd	Associative matrix key/value containing the challenges to verify the integrity of the file
chunkSize	1st	Size in bytes of each of the 4096 file fractions.
clientAddress	1st	Address that identifies the file owner (client) in the BN
cyclesFinalized	2nd	Associative matrix key/value containing the identifiers of the verification cycles already completed, that is, those whose 256 challenges that compose the cycle have already been answered
fileId	1st	Hash used to identify the stored file and verify its integrity when uploading or downloading
integrityCheckAgreementDue	1st	End date of the current integrity verification contract
integrityCheckServiceAddress	1st	Address that identifies the ICS hired to monitor the file integrity in the BN
requestedChallenges	2nd	Associative matrix key/value containing challenge requests for audit purposes
storageLimitDate	1st	End date of the file storage period contracted with the CSS
storageServiceAddress	1st	Address that identifies the CSS hired to store the file in the BN
totalBlocks	1st	Number of data blocks that will be used to verify the file integrity generated by the client before storing the file in the cloud
trustContract	1st	SSTMC instance responsible for managing trust in the CSS that stores the file

**Table 4:** Functions of CFSMC methods and the roles that can perform them.

Method	Group	Function	Execution
changeIntegrityCheckService	2nd	Receive and replace the address in the BN of the ICS hired to perform the file integrity monitoring in the attribute integrityCheckServiceAddress	Client (file owner)
getChallenge	3rd	Return the data for a challenge registered by the ICS/Client and stored as an element of the attribute challenges	Public
getChallengeStatus	3rd	Return the challenge status according to the receipt of the response and its validation (pending, success, or failure) obtained from the respective element stored in the attribute challenges	Public
getPendingChallenges	3rd	Return a list containing the identifiers of the pending challenges stored in the attribute challenges	Public
getTotalFailedChallenges	3rd	Return the number of challenges registered with the situation “failed” in the attribute challenges	Public
getTotalStoredBlocks	1st	Return the amount of VHs of the data blocks that have already been inserted by the client in the CFSMC (number of elements stored in the attribute blocks)	Public
getTrustManagementContract	1st	Return the access address in the BN of the CFSMC instance chosen by the client to manage the trust in the CSS that stored the file, whose object is stored in the attribute trustContract	Public
get{ChunkSize, ClientAddress, FileId, StorageLimitDate, TotalBlocks}	1st	Return the value stored in the respective attribute (chunkSize, clientAddress, fileId, storageLimitDate, totalBlocks)	Public
get{IntegrityCheckAgreement- Due, IntegrityCheckServiceAddress, StorageServiceAddress}	2nd	Return the value stored in the respective attribute (integrityCheckAgreementDue, integrityCheckServiceAddress, storageServiceAddress)	Public
insertBlock	3rd	Receive and store a set of VHs of data blocks in the attribute blocks	Client (file owner)
isAccepted	1st	Inform if this CFSMC instance has already received acceptance from the CSS in the chosen SSTMC instance	Public
isCycleFinalized	3rd	Inform if a particular verification cycle has already been completed, i.e., if its identifier is on the list of elements stored in the attribute cyclesFinalized	Public
isReady	1st	Inform if the CFSMC is ready to be audited, i.e., if the number of VHs inserted (number of elements in the attribute blocks) is equal to the number of data blocks generated by the Client (attribute totalBlocks)	Public
replyChallenge	3rd	Receive and validate the responses to the challenges, updating the respective element of the attribute challenges with the response and the result of the validation.	CSS hired
requestChallenge	3rd	Receive and store an audit challenge request in the attribute requestedChallenges	CSS hired
setIntegrityCheckAgreementDue	2nd	Receive and store the end date of the current integrity verification contract in the attribute integrityCheckAgreementDue	Client (file owner)
submitChallenge	3rd	Receive and store the challenges for verifying the file integrity in the attribute challenges	Client and ICS hired
verifyChallenge	3rd	Perform the validity check of the pending challenge stored as an element of the attribute challenges, changing its situation to failure if the challenge exceeded the maximum waiting for a response period (expired).	ICS hired

**Table 5:** Summary of time period spent preparing files for storage.

Size of	Storage Time	Average of Time Spent to			
File	(Years)	Encrypt File	Hash File	Hash Data Blocks	Save Database
52 MB	1	00:00:02.807	00:00:00.395	00:00:05.733	00:00:07.988
196 MB	2	00:00:11.796	00:00:01.213	00:00:41.569	00:00:11.670
243 MB	3	00:00:14.159	00:00:01.317	00:01:16.001	00:00:12.999
593 MB	4	00:00:29.728	00:00:03.715	00:04:07.053	00:00:15.737
750 MB	5	00:00:38.259	00:00:03.829	00:06:32.848	00:00:17.567
1 GB	10	00:00:52.907	00:00:05.564	00:19:10.344	00:00:27.924
2 GB	15	00:01:54.326	00:00:11.250	00:54:08.700	00:00:36.470
5 GB	20	00:05:16.666	00:00:48.958	03:50:55.159	00:00:43.656
10 GB	25	00:08:11.177	00:01:11.778	11:00:34.356	00:00:53.099

**Table 6:** Results of validation session per CSS.

File Corrupted	Failure Identification Day		
	CSS 1	CSS 2	CSS 3
1st	7th	8th	1st
2nd	15th	9th	16th
3rd	24th	9th	43th
4th	27th	15th	45th
5th	31th	17th	45th
6th	36th	23th	52th
7th	40th	24th	52th
8th	40th	28th	57th
9th	45th	38th	60th
10th	48th	43th	61th
11th	49th	45th	63th
12th	52th	47th	63th
13th	56th	51th	63th
14th	56th	52th	64th
15th	65th	62th	66th
16th	66th	63th	70th
17th	68th	66th	71th
18th	69th	69th	78th
19th	69th	69th	82th
20th	74th	73th	85th
21th	75th	74th	87th
22th	78th	76th	88th
23th	81th	76th	89th
24th	82th	81th	89th
25th	88th	82th	92th
26th	88th	84th	95th
27th	92th	86th	98th

**Table 7:** Summary of results by file size and storage time.

Size of File	Storage Time	Days to Identify the Integrity Violation		
		Minimum	Maximum	Average
50 MB	1 year	1	74	46.8
5 GB	20 years	15	98	47.6
750 MB	5 years	7	81	48.8
200 MB	2 years	15	92	55.7
1 GB	10 years	24	82	60.4
600 MB	4 years	8	88	63.0
250 MB	3 years	9	88	63.7
2 GB	15 years	24	95	65.0
10 GB	25 years	45	92	66.8

**Table 8:** Summary of the results of this validation.

CSS	Checked Files	Days to Identify the Integrity Violation		
		Minimum	Maximum	Average
1	27	7	92	55.1
2	27	8	86	50.7
3	27	1	98	66.5
General	81	1	98	57.4

## Data Availability

Not applicable.

## References

[1] Brazil (2001). Medida Provisória n° 2.200-2, de 24 de agosto de 2001. Diário Oficial [da] República Federativa do Brasil.

[2] United States of America Public Law 106-229: Electronic Signatures in Global and National Commerce Act. https://www.govinfo.gov/content/pkg/PLAW-106publ229/pdf/PLAW-106publ229.pdf.

[3] Brazil (2001). Arquivo Nacional. Conselho Nacional de Arquivos. Classificação, Temporalidade e Destinação de Documento de Arquivo.

[4] United Kingdom The Money Laundering, Terrorist Financing and Transfer of Funds (Information on the Payer) Regulations. https://www.legislation.gov.uk/uksi/2017/692.

[5] Amaral D.M., Gondim J.J., Albuquerque R.D.O., Orozco A.L.S., Villalba L.J.G. (2019). Hy-SAIL. IEEE Access.

[6] Tang X., Huang Y., Chang C.C., Zhou L. (2019). Efficient Real-Time Integrity Auditing With Privacy-Preserving Arbitration for Images in Cloud Storage System. IEEE Access.

[7] Zhao H., Yao X., Zheng X., Qiu T., Ning H. (2019). User stateless privacy-preserving TPA auditing scheme for cloud storage. J. Netw. Comput. Appl..

[8] Wang F., Xu L., Wang H., Chen Z. (2018). Identity-based non-repudiable dynamic provable data possession in cloud storage. Comput. Electr. Eng..

[9] Jeong J., Joo J.W.J., Lee Y., Son Y. (2019). Secure cloud storage service using bloom filters for the internet of things. IEEE Access.

[10] Pinheiro A., Dias Canedo E., de Sousa Junior R., de Oliveira Albuquerque R., García Villalba L., Kim T.H. (2018). Security Architecture and Protocol for Trust Verifications Regarding the Integrity of Files Stored in Cloud Services. Sensors.

[11] Pinheiro A., Canedo E.D., De Sousa R.T., Albuquerque R.D.O. (2020). Monitoring File Integrity Using Blockchain and Smart Contracts. IEEE Access.

[12] Google Google Cloud Platform—Cloud Storage. https://cloud.google.com/storage.

[13] Amazon Amazon Simple Storage Service (Amazon S3). http://aws.amazon.com/pt/s3.

[14] Microsoft Microsoft Azure—Storage. https://azure.microsoft.com/en-us/services/storage.

[15] Walport M. (2016). Distributed Ledger Technology.

[16] Hill B., Chopra S., Valencourt P., Prusty N. (2018). Blockchain Developer’s Guide.

[17] Sunyaev A. (2020). Distributed ledger technology. Internet Computing.

[18] Lange M., Leiter S.C., Alt R., Di Ciccio C., Gabryelczyk R., García-Bañuelos L., Hernaus T., Hull R., Štemberger M.I., Kő A., Staples M. (2019). Defining and Delimitating Distributed Ledger Technology. Business Process Management: Blockchain and Central and Eastern Europe Forum. BPM 2019.

[19] Fernando D., Ranasinghe N., Di Ciccio C., Gabryelczyk R., García-Bañuelos L., Hernaus T., Hull R., Štemberger M.I., Kő A., Staples M. (2019). Permissioned Distributed Ledgers for Land Transactions; A Case Study. Business Process Management: Blockchain and Central and Eastern Europe Forum. BPM 2019.

[20] Maull R., Godsiff P., Mulligan C., Brown A., Kewell B. (2017). Distributed ledger technology. Strateg. Chang..

[21] Ølnes S., Ubacht J., Janssen M. (2017). Blockchain in government. Gov. Inf. Q..

[22] Alharby M., van Moorsel A. (2017). Blockchain-based smart contracts. Comput. Sci. Inf. Technol..

[23] Abeyratne S.A., Monfared R.P. (2016). Blockchain ready manufacturing supply chain using distributed ledger. Int. J. Res. Eng. Technol..

[24] Cong L.W., He Z. (2019). Blockchain disruption and smart contracts. Rev. Financ. Stud..

[25] Wüst K., Gervais A. Do you need a blockchain?. Proceedings of the 2018 Crypto Valley Conference on Blockchain Technology (CVCBT).

[26] Christidis K., Devetsikiotis M. (2016). Blockchains and smart contracts for the internet of things. IEEE Access.

[27] Bitcoin.org Bitcoin. https://bitcoin.org/.

[28] Nakamoto S. Bitcoin: A Peer-to-Peer Electronic Cash System. https://bitcoin.org/bitcoin.pdf.

[29] Ethereum Foundation Ethereum. https://ethereum.org/.

[30] Buterin V. (2013). Ethereum White Paper: A Next Generation Smart Contract & Decentralized Application Platform. http://bitpaper.info/paper/5634472569470976.

[31] Kraft D., Castellucci R., Rand J., Roberts B., Bisch J., Colosimo A., Conrad P., Bodiwala A., Dam L. Namecoin: Decentralize All the Things. https://www.namecoin.org/.

[32] Kalodner H.A., Carlsten M., Ellenbogen P., Bonneau J., Narayanan A. An Empirical Study of Namecoin and Lessons for Decentralized Namespace Design. Proceedings of the Workshop on the Economics of Information Security (WEIS).

[33] Coin Sciences Ltd Multichain: Enterprise Blockchain. That Actually Works. https://www.multichain.com/.

[34] Electric Coin Co Zcash. https://z.cash/.

[35] The Monero Project Monero: A Private Digital Currency. https://www.getmonero.org/.

[36] Noether S., Noether S. (2014). Monero Is Not That Mysterious. https://web.getmonero.org/ru/resources/research-lab/pubs/MRL-0003.pdf.

[37] The Linux Foundation Hyperledger: Advancing Business Blockchain Adoption through Global Open Source Collaboration. https://www.hyperledger.org/.

[38] Androulaki E., Barger A., Bortnikov V., Cachin C., Christidis K., De Caro A., Enyeart D., Ferris C., Laventman G., Manevich Y. Hyperledger fabric: A distributed operating system for permissioned blockchains. Proceedings of the European Conference on Computer Systems (EuroSys).

[39] Maltseva D. 10 Most Popular & Promising Blockchain Platforms. https://dev.to/dianamaltseva8/10-most-popular--promising-blockchain-platforms-djo.

[40] Subramanian B. (2019). Top 5 Use Cases and Platforms of Blockchain Technology.

[41] Sonee S. (2020). 5 Best Platform for Building Blockchain-Based Applications.

[42] Sajana P., Sindhu M., Sethumadhavan M. (2018). On blockchain applications. Int. J. Pure Appl. Math..

[43] Ethereum Foundation Solidity. https://solidity.readthedocs.io/.

[44] Yuan R., Xia Y.B., Chen H.B., Zang B.Y., Xie J. (2018). Shadoweth: Private smart contract on public blockchain. J. Comput. Sci. Technol..

[45] Valenta M., Sandner P. (2017). Comparison of Ethereum, Hyperledger Fabric and Corda.

[46] Wood G. Ethereum: A Secure Decentralised Generalised Transaction Ledger. https://files.gitter.im/ethereum/yellowpaper/VIyt/Paper.pdf.

[47] Vo-Cao-Thuy L., Cao-Minh K., Dang-Le-Bao C., Nguyen T.A. Votereum: An Ethereum-Based E-Voting System. Proceedings of the 2019 IEEE-RIVF International Conference on Computing and Communication Technologies (RIVF).

[48] Crafa S., Di Pirro M., Zucca E. (2019). Is solidity solid enough?. Financial Cryptography and Data Security.

[49] Kiayias A., Zindros D. (2019). Proof-of-work sidechains. Financial Cryptography and Data Security.

[50] Maymounkov P. (2002). Online Codes.

[51] Pugh W. (1990). Skip lists. Commun. ACM.

[52] Erway C.C., Küpçü A., Papamanthou C., Tamassia R. (2015). Dynamic provable data possession. ACM Trans. Inf. Syst. Secur..

[53] Shacham H., Waters B., Pieprzyk J. (2008). Compact proofs of retrievability. Advances in Cryptology. ASIACRYPT 2008.

[54] Boneh D., Buhler J.P. (1998). The decision diffie-hellman problem. Algorithmic Number Theory. ANTS 1998.

[55] Diffie W., Hellman M. (1976). New directions in cryptography. IEEE Trans. Inf. Theory.

[56] Galindo D., Garcia F.D., Preneel B. (2009). A Schnorr-like lightweight identity-based signature scheme. Progress in Cryptology. AFRICACRYPT 2009.

[57] Bloom B.H. (1970). Space/time trade-offs in hash coding with allowable errors. Commun. ACM.

[58] Zhang Y., Xu C., Lin X., Shen X.S. (2019). Blockchain-based public integrity verification for cloud storage against procrastinating auditors. IEEE Trans. Cloud Comput..

[59] Boneh D., Lynn B., Shacham H. (2004). Short signatures from the Weil pairing. J. Cryptol..

[60] Rahalkar C., Gujar D. (2019). Content Addressed P2P File System for the Web with Blockchain-Based Meta-Data Integrity. Proceedings of the 2019 International Conference on Advances in Computing, Communication and Control (ICAC3).

[61] Meroni G., Plebani P., Vona F., Hildebrandt T., van Dongen B., Rolinger M., Mendling J. (2019). Trusted artifact-driven process monitoring of multi-party business processes with blockchain. Business Process Management: Blockchain and Central and Eastern Europe Forum. BPM 2019.

[62] Christen P., Ranbaduge T., Schnell R. (2020). Linking Sensitive Data.

[63] Wang S., Tang X., Zhang Y., Chen J. (2019). Auditable Protocols for Fair Payment and Physical Asset Delivery Based on Smart Contracts. IEEE Access.

[64] Consensys Ethereum Smart Contract Security Best Practices. https://consensys.github.io/smart-contract-best-practices/.

